# Reciprocal activation of antigen-presenting cells and CAR T cells triggers a widespread endogenous anti-tumor immune response through sustained high-level IFN*γ* production

**DOI:** 10.20892/j.issn.2095-3941.2023.0324

**Published:** 2023-11-06

**Authors:** Yelei Guo, Chuan Tong, Zhiqiang Wu, Yuting Lu, Yao Wang, Weidong Han

**Affiliations:** Department of Bio-therapeutic, the First Medical Center, Chinese PLA General Hospital, Beijing 100853, China

Adoptive cell transfer (ACT) using chimeric antigen receptor (CAR) modified T cells and T cell receptor (TCR) engineered T cells has shown therapeutic efficacy in cancer treatment^1,2^. CAR T cells are widely applicable to tumor patients because of their ability to directly identify tumor cells in an MHC-independent manner. Recent studies have shown that antigen escape, including antigenic heterogeneity, wherein not all tumor cells express the targeted antigen, as well as antigen loss, are key challenges in CAR T treatment of tumors, particularly solid tumors^3^. Indeed, antigen escape has been reported in patients with metastatic pancreatic carcinoma treated with CAR T cells redirected against epidermal growth factor receptor (EGFR) and patients with relapsed glioblastoma (GBM) treated with anti-EGFRvIII CAR T cells^4,5^. CAR T cells have successfully eliminated tumors expressing the target antigen but not antigen-negative tumors. These observations highlight the need for novel strategies to avoid immune escape through antigenic heterogeneity and antigen loss.

## Antigen spreading elicited by adoptive cell transfer

In addition to the use of dual-specific T cells or cocktail treatment with CAR T cells to address tumor antigenic heterogeneity and antigen loss^6,7^, accumulating evidence supports the emerging concept of triggering the endogenous immune system to establish an effective anti-tumor response. Antigen spreading (AS), a mode of activation of the endogenous immune system, is elicited after primary therapy-induced tumor lysis and results in the release of secondary tumor antigens other than the CAR T cell targeted epitope. Subsequently, antigen-presenting cells (APCs), such as dendritic cells (DCs), take up these secondary antigens and induce an endogenous T cell response, thereby eliminating tumor cells^8^. In the context of ACT, approaches using CAR T cells to induce endogenous T cell responses may be a promising strategy to overcome antigen escape.

In fact, AS into the support anti-tumor response of CAR T cells has been demonstrated in recent studies. In the July 2023 issue of *Cell*, Ma et al.^3^ reported that vaccine-boosting increases the metabolic fitness of CAR T cells and facilitates communication between CAR T cells and the endogenous immune system, thus inducing and maintaining an AS response *in vivo*. In that study, an amph-ligand-based vaccine containing a ligand for a selected CAR linked to a hydrophobic phospholipid tail *via* a polyethylene glycol spacer was designed. After co-injection of the amph-ligand-based vaccine with a suitable adjuvant—such as the stimulator of interferon genes (STING) agonist cyclic di-GMP, the Toll-like receptor (TLR) 7/8 agonist Resiquimod, or the TLR9 agonist CpG—the amph-ligands bind albumin in the interstitial fluid, migrate to draining lymph nodes (dLNs), and are transferred to cell membranes and subsequently decorate the surfaces of DCs and macrophages. DCs are simultaneously activated by the co-injected adjuvant in dLNs, thus increasing costimulatory receptor expression and cytokine production. Subsequently, CAR T cells, after encountering ligand-decorated, activated DCs, are stimulated to shift their metabolism toward oxidative phosphorylation, in a manner mimicking the activation of natural T cells. Consequently, CAR T cells expand and show enhanced effector function. In murine syngeneic tumor models, DCs are recruited by vaccine-boosting of CAR T cells into the tumor mass, thus augmenting antigen uptake and processing capacity, and magnifying the priming of endogenous T cell anti-tumor responses. This process may eliminate antigenic heterogeneous tumor cells through AS of endogenous T cells. Mechanistically, CAR T cell derived IFNγ after vaccine boosting plays a critical role in AS initiation. Reciprocally, IFNγ stimulated DCs enhance the secretion of IL-12, which in turn further amplifies autocrine IFNγ secretion by CAR T cells. The concept of IFNγ signaling promoting endogenous T cell immunity has also been confirmed in syngeneic GBM transplanted murine models treated with anti-IL13Ra2 CAR T cells^9^. CAR T cells derived from IFNγ−/− mice exhibit poorer anti-tumor activity *in vivo* than CAR T cells generated from wild type (WT) mice, thus indicating that IFNγ deficiency dampens the anti-tumor effects of CAR T cells *in vivo*.

To achieve AS in the setting of ACT to compete against antigen escape of heterogeneous tumor cells, several studies have focused on CAR T cells engineered with additional immunostimulatory factors, such as FLT3L^10^ and CD40L^11^. These studies have provided evidence that AS increases anti-tumor activity.

The conditions associated with the production of high levels of IFNγ by CAR T cells include (1) activation of APCs by a suitable adjuvant; (2) CAR ligand decoration or presentation on APCs; (3) CAR T cells encountering these activated and decorated APCs in dLNs *via* the CAR-ligand axis and costimulatory factor-receptor axis, and being activated in a manner mimicking the activation of natural T cells; and (4) that IL-12 secretion from activated DCs stimulating CAR T cells to further maintain high IFNγ expression. Moreover, the activation of APCs plays a crucial role in AS in cancer treatments, such as ACT. On this basis, AS can be elicited in the setting of tumor therapy with CAR T cells.

## IFNγ: a master initiator triggering pleiotropically endogenous anti-tumor immunity

IFNγ, a key proinflammatory cytokine produced by activated CAR T cells, plays an important role in promoting endogenous immune cell infiltration to tumor masses and subsequent reprogramming^3,9^. For example, IFNγ promotes the recruitment and activation of cytotoxic T cells, and polarization of CD4^+^ T cells into T helper (Th) 1 cells. In addition, amph-ligand based vaccine boosted CAR T cell-derived IFNγ upregulates DC-recruiting chemokine expression in tumors, thereby increasing DC infiltration and activation, and eliciting an AS response that eliminates CAR-target antigen-negative tumor cells^3^. Moreover, IFNγ, together with IL-12, secreted by activated DCs, promotes the differentiation of naïve T cells into Th1 cells^12,13^.

In addition, IFNγ has been reported to activate macrophages^14,15^. Alizadeh et al.^9^ have shown that IFNγ produced by anti-IL13Ra2 CAR T cells from a mouse model of GBM endows intratumoral myeloid cells with anti-tumor activity. The frequency of MHC I^+^/MHC II^+^ and CD86^+^ macrophage activation is significantly upregulated in tumor bearing mice treated with CAR T^WT^ cells rather than CAR T^IFNγ−/−^ cells; nevertheless, the frequency of intratumoral M2-type macrophages is higher in mice treated with CAR T^IFNγ−/−^ cells^9^. Another recent study has indicated that IFNγ derived from CD4^+^ T cells either directly kills IFNγ-sensitive tumor cells or induces intratumoral myeloid cells to become iNOS-expressing tumoricidal types^16^. A highly specific iNOS inhibitor has been found to abrogate the anti-tumor activity of a CD4 based treatment (involving co-infusion of CD4^+^ TCR T cells, a vaccine comprising the recombinant adenovirus AD-PT encoding the TCR-target antigen, and innate stimulators that activate innate immunity through TLR3 and TLR9) in mice bearing MHC I/II-deficient IFNγ-unresponsive melanoma cells preconditioned with cyclophosphamide. These results indicated that IFNγ derived from CAR T cells or TCR T cells activates myeloid cells to eliminate tumor cells.

**Figure 1:**
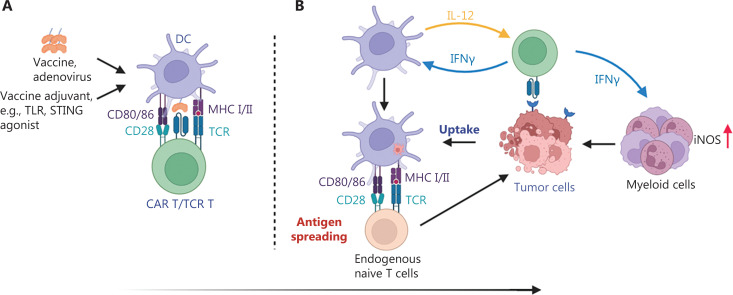
Schematic overview of reciprocal activation of CAR T cells with antigen-presenting cells triggering a widespread endogenous anti-tumor immune response. (A) CAR/TCR ligand-decorated DCs activated through vaccine or adenovirus, and vaccine adjuvants such as STING or TLR agonists, stimulate CAR T/TCR T cell activation and expansion in lymph nodes. (B) Activated CAR T/TCR T cells migrate into the tumor mass and kill CAR/TCR-targeted antigen-positive tumor cells. CAR T/TCR T cells secrete IFNγ, thus inducing DC recruitment and activation, and secondary tumor antigen uptake, and activating antigen spreading. Meanwhile, IFNγ stimulates myeloid cell polarization into iNOS-expressing tumoricidal myeloid cells. Created with Biorender.com.

Thus, CAR T cell-derived IFNγ plays a crucial role in promoting endogenous immunity, including AS and myeloid cell activation, and further prevents tumor antigenic heterogeneity, as well as antigen loss mediated tumor escape and recurrence (**Figure 1**).

While strengthening endogenous immunity, IFNγ does not affect tumor angiogenesis^3^ but promotes PD-L1 expression in tumor cells, thus leading to lymphocyte dysfunction through the PD-1-PD-L1 axis^17,18^. Multiple factors, such as expression levels of IFNγ and its receptor on tumor cells, suggest that IFNγ mediates the expression of PD-L1 in tumors^17–20^. Whether enhanced endogenous immunity derived from IFNγ secreted by CAR T cells counteracts lymphocyte dysfunction remains to be further confirmed.

## Clinical translation

Tumor-associated antigen is commonly used as a target for CAR T cells in solid tumors, but its heterogeneous expression impairs the efficiency of CAR T cell therapy. Effective approaches to combat antigenic heterogeneity and antigen loss are highly valuable in increasing the efficacy of CAR T cells in solid tumors. Indeed, a variety of approaches have been reported to reinforce the efficacy of CAR T cells against solid tumors, by potentiating CAR T cells, other host immune cells, or both^3,10,11^. Observations from recent studies suggest that the activation of endogenous immunity may extend the anti-tumor efficacy of CAR T cells by overcoming heterogeneous antigen expression on tumor cells.

Promoting endogenous immunity to enhance the anti-tumor activity of CAR T cells is anticipated in clinical trials. AS in CAR T cell therapy rarely has adequate treatment effects, possibly because the activation of DCs is insufficient for the initial cross-activation of endogenous T cells and effector T cell recruitment into the tumor microenvironment. Over-expression of FLT3L^10^ and CD40L^11^ on CAR T cells, and co-infusion with vaccine adjuvants such as STING-ligand, TLR 7/8 agonist, and TLR9 agonist^3^, may induce activation of DCs, thereby stimulating the endogenous immune system to assist in CAR T cell anti-tumor activity. Furthermore, the use of armed CAR T cells with sustained expression of IFNγ in an antigen-dependent manner to activate endogenous immunity and elicit tumor destruction is also valuable in tumor treatment.

## Summary

ACT with CAR T cells has been highly successful in the treatment of relapsed/refractory CD19^+^ B cell acute lymphoblastic leukemia and lymphoma. Several issues, including limited tumor infiltration, functionality, and persistence, limit the benefits of CAR T cell therapy for patients with solid tumors. Although a variety of strategies have recently been developed to enhance the anti-tumor activity of CAR T cells for solid tumors, pre-existing antigenic heterogeneity and antigen loss during treatment greatly restrain CAR T cell efficacy. Accumulating evidence highlights that promoting endogenous immunity, including AS and myeloid cell activation, may enhance the overall treatment response in the ACT setting. Although this modality is currently in the proof-of-concept stage, it may support a wide range of clinical applications in patients with solid tumors.

## References

[1] Han L, Zhou J, Li L, Zhou K, Zhao L, Zhu X (2021). Culturing adequate CAR-T cells from less peripheral blood to treat B-cell malignancies. Cancer Biol Med.

[2] Li P, Liu Y, Liang Y, Bo J, Gao S, Hu Y (2023). 2022 Chinese expert consensus and guidelines on clinical management of toxicity in anti-CD19 chimeric antigen receptor T-cell therapy for B-cell non-hodgkin lymphoma. Cancer Biol Med.

[3] Ma L, Hostetler A, Morgan DM, Maiorino L, Sulkaj I, Whittaker CA (2023). Vaccine-boosted CAR T crosstalk with host immunity to reject tumors with antigen heterogeneity. Cell.

[4] O’Rourke DM, Nasrallah MP, Desai A, Melenhorst JJ, Mansfield K, Morrissette JJD (2017). A single dose of peripherally infused EGFRvIII-directed car T cells mediates antigen loss and induces adaptive resistance in patients with recurrent glioblastoma. Sci Transl Med.

[5] Liu Y, Guo YL, Wu ZQ, Feng KC, Tong C, Wang Y (2020). Anti-EGFR chimeric antigen receptor-modified T cells in metastatic pancreatic carcinoma: a phase I clinical trial. Cytotherapy.

[6] Tong C, Zhang YJ, Liu Y, Ji XY, Zhang WY, Guo YL (2020). Optimized tandem CD19/CD20 car-engineered T cells in refractory/relapsed B-cell lymphoma. Blood.

[7] Feng KC, Guo YL, Liu Y, Dai HR, Wang Y, Lv HY (2017). Cocktail treatment with EGFR-specific and CD133-specific chimeric antigen receptor-modified T cells in a patient with advanced cholangiocarcinoma. J Hematol Oncol.

[8] Gulley JL, Madan RA, Pachynski R, Mulders P, Sheikh NA, Trager J (2017). Role of antigen spread and distinctive characteristics of immunotherapy in cancer treatment. J Natl Cancer Inst.

[9] Alizadeh D, Wong RA, Gholamin S, Maker M, Aftabizadeh M, Yang X (2021). IFNγ is critical for CAR T cell-mediated myeloid activation and induction of endogenous immunity. Cancer Discov.

[10] Lai J, Mardiana S, House IG, Sek K, Henderson MA, Giuffrida L (2020). Adoptive cellular therapy with T cells expressing the dendritic cell growth factor Flt3L drives epitope spreading and antitumor immunity. Nat Immunol.

[11] Kuhn NF, Lopez AV, Li X, Cai W, Daniyan AF, Brentjens RJ (2020). CD103(+) cDC1 and endogenous CD8(+) T cells are necessary for improved CD40L-overexpressing CAR T cell antitumor function. Nat Commun.

[12] Schulz EG, Mariani L, Radbruch A, Hofer T (2009). Sequential polarization and imprinting of type 1 T helper lymphocytes by interferon-γ and interleukin-12. Immunity.

[13] Caspi RR (2002). Th1 and th2 responses in pathogenesis and regulation of experimental autoimmune uveoretinitis. Int Rev Immunol.

[14] Duluc D, Corvaisier M, Blanchard S, Catala L, Descamps P, Gamelin E (2009). Interferon-γ reverses the immunosuppressive and protumoral properties and prevents the generation of human tumor-associated macrophages. Int J Cancer.

[15] Jansen CS, Prokhnevska N, Master VA, Sanda MG, Carlisle JW, Bilen MA (2019). An intra-tumoral niche maintains and differentiates stem-like CD8 T cells. Nature.

[16] Kruse B, Buzzai AC, Shridhar N, Braun AD, Gellert S, Knauth K (2023). CD4(+) T cell-induced inflammatory cell death controls immune-evasive tumours. Nature.

[17] Abiko K, Matsumura N, Hamanishi J, Horikawa N, Murakami R, Yamaguchi K (2015). IFN-γ from lymphocytes induces PD-L1 expression and promotes progression of ovarian cancer. Br J Cancer.

[18] Bellucci R, Martin A, Bommarito D, Wang K, Hansen SH, Freeman GJ (2015). Interferon-γ-induced activation of JAK1 and JAK2 suppresses tumor cell susceptibility to NK cells through upregulation of PD-L1 expression. Oncoimmunology.

[19] Benci JL, Johnson LR, Choa R, Xu Y, Qiu J, Zhou Z (2019). Opposing functions of interferon coordinate adaptive and innate immune responses to cancer immune checkpoint blockade. Cell.

[20] Chen S, Crabill GA, Pritchard TS, McMiller TL, Wei P, Pardoll DM (2019). Mechanisms regulating PD-L1 expression on tumor and immune cells. J Immunother Cancer.

